# Increasing physical activity levels following treatment for cervical cancer: an intervention mapping approach

**DOI:** 10.1007/s11764-021-01058-y

**Published:** 2021-05-26

**Authors:** Nessa Millet, Hilary J. McDermott, Esther L. Moss, Charlotte L. Edwardson, Fehmidah Munir

**Affiliations:** 1 School of Sport, Exercise and Health Sciences, National Centre for Sport and Exercise Medicine, Loughborough University, Loughborough, LE11 3TU UK; 2 Diabetes Research Centre, University of Leicester, Leicester, LE54PW UK; 3 NIHR Leicester Biomedical Research Centre, Leicester, LE3 9QP UK; 4 Leicester Cancer Research Centre, University of Leicester, Leicester, LE2 7Lx UK

**Keywords:** Gynaecological oncology, Physical activity, Intervention mapping, Quality of life

## Abstract

**Purpose:**

The purpose of this study was to utilise the intervention mapping (IM) protocol as a framework with which to develop an intervention underpinned by relevant behaviour change theory to promote physical activity (PA) following treatment for cervical cancer.

**Methods:**

The six steps of the IM protocol were followed. A qualitative semi-structured interview study and a rapid review of the literature were conducted along with the development of a logic model of the problem and a logic model of change to inform intervention development.

**Results:**

An intervention was developed which aims to increase PA levels following treatment for cervical cancer, tailored to address key findings from the IM needs assessment. These include embedding behavioural and social strategies that help participants to overcome perceived barriers to PA participation; goal setting strategies to gradually increase PA levels with a view of reaching relevant PA guidelines for cancer survivors and feedback to encourage self-assessment of well-being and PA capability.

**Conclusion:**

This study maps the development of a novel PA intervention for those who have been treated for cervical cancer. The use of a systematic development framework was necessary as little insight exists regarding PA preferences after treatment for cervical cancer.

**Implications for Cancer Survivors:**

PA behaviour is associated with positive physical and psychological health outcomes for cancer survivors. Optimising targeted promotion of PA behaviour following treatment for cervical cancer may result in an enhanced survivorship experience through increased PA behaviour and improved quality of life (QOL).

**Supplementary Information:**

The online version contains supplementary material available at 10.1007/s11764-021-01058-y.

## Background

The most recent data suggests that there are on average 3200 cases of cervical cancer diagnosed each year [1]. Survival rate has improved over the last 40 years with documented increases in the number of cervical cancer survivors beyond 5 years or more (60%) [1]. Despite survivors being at risk of experiencing several health and lifestyle related issues [2, 3], very few lifestyle interventions exist which are specifically aimed at improving QOL following cervical cancer and its associated treatments. Therefore, an increased focus on survivorship and interventions to enhance recovery after cervical cancer are warranted.

Navigating the recovery period to regain a sense of normality following treatment for cervical cancer is perceived as challenging [4]. This can be due to a myriad of psychological, physical and social impacts of treatment-related changes [5]. Research suggests that individuals treated for cervical cancer, particularly with radiotherapy, tend to exhibit worse QOL scores compared to those treated for other gynaecological tumours [6]. Premature menopause is a particularly disabling after-effect of treatment, predominantly for those treated at a young age due to the comorbid negative symptomology that may be experienced as a result (e.g. osteopenia and vasomotor symptoms [7]). Additionally, long-term psychological distress is common [8], often due to sexual dysfunction, fear and anxiety related to cancer recurrence and a loss of fertility [9]. Previous explorations have demonstrated a need for psychosocial intervention to improve the survivorship experience [5].

PA has been consistently shown to have positive psychological benefits after cancer [10], yet studies frequently highlight that survivors report low levels of PA up to 3 years after treatment for gynaecological cancers [11]. Meta-analysis data indicates that PA interventions are widely investigated and are a safe way to improve QOL [12]. However, the body of literature in this area lacks diversity in tumour type with most studies conducted among breast cancer survivors [13]. The limited existing evidence following cervical cancer suggests that PA can result in improvements in fatigue [14] and sexual functioning [15]. Further support is needed to strengthen findings and to determine preferable modalities and durations specifically in this population. Therefore, the current study aimed to design a behaviour change intervention to increase PA levels following treatment for cervical cancer.

The most effective behaviour change interventions are those which explicitly link theory to the components of an intervention [16]. It has been strongly suggested that this becomes common practice in order to maximise the effectiveness of interventions and the potential for refinement and progression through robust evaluation [17]. Considering this, the development of the current intervention followed the IM protocol which is a six-step systematic framework for intervention development [18] and which has been previously used in cancer populations [19]. The IM protocol allows the integration of experience and insight from the target population whilst also prioritising relevant behaviour change theories to inform the intervention components (e.g. [19]).

## Method

Firstly, a planning group with relevant experience was established. The planning group consisted of a PhD student specialising in exercise psychology with 2 years of experience in PA intervention design (NM). Three of the authors (FM, HM, CE) together have at least 15 years of experience in intervention design and implementation research. Three of the authors have also worked specifically with cancer groups in research and practice (EM, 18 years; FM; HM, at least 15 years). Secondly, in line with guidance by the National Institute for Health Research (NIHR) [20], two patient and public involvement (PPI) groups were established over the course of the research. The initial discussion with a group of six patients treated for cervical cancer enabled the identification of unmet needs and guided initial research aims. A second, smaller group of three patients treated for cervical cancer met with NM as a group every 6 months and individually to act as advisors throughout and to ensure that aspects of the research were user-friendly. The planning group worked through the six steps of the IM protocol as follows, which are presented in line with relevant previous research [21, 22]:

### Step 1: Needs assessment

First, the planning group developed the logic model of the problem (i.e. low PA participation in the target group) based on their expertise and their knowledge of the relevant scientific literature [18]. The logic model was then refined after undertaking a needs assessment which consisted of: (1) a rapid review of the relevant literature and (2) semi-structured interviews with women focusing on their experience following treatment for cervical cancer and their PA preferences.

#### Rapid review of the literature

A scientific literature review was conducted by NM to identify published studies on cervical cancer survivorship and PA. Given the scarcity of literature in the area representing cervical cancer survivors, research in other gynaecological cancer populations was also considered; however, only studies which recruited gynaecological cancer survivors as their sole population were reported. The review utilised several search engines (Google Scholar, PubMed, Web of Science) to identify (a) PA intervention studies in gynaecological cancer survivors (including some representation of cervical cancer) and PA preferences of gynaecological cancer survivors (b) theoretical underpinnings of relevant PA interventions. The search terms used can be found in Appendix A.

#### Interviews with women who have been treated for cervical cancer

Ethical approval was gained from the Loughborough University Ethics Sub-Committee to conduct interviews with those treated for cervical cancer aged between 18 and 65 years. Participants were purposefully recruited via charities, recruitment posters and social media. Those treated for pre-invasive cervical lesions only and those who were unable to partake in PA due to an issue unrelated to their cancer were excluded. Informed consent was obtained prior to participation. Ten participants were interviewed either face to face or over the telephone. A semi-structured interview schedule was followed, which asked questions on what types of movements participants perceived themselves capable of doing since treatment, if, and how much, they were typically active and their perceived challenges and facilitators to taking part in PA (e.g. “If you are someone who would like to be more physically active, what is stopping you now?”). Interviews were digitally recorded and transcribed verbatim manually. Interviews were analysed manually using template analysis, a form of thematic analysis [23] which includes a large degree of structure in the analysis process by utilising a coding template (Appendix B).

### Step 2: Identification of outcomes, performance objectives and change objectives

In Step 2, a logic model of change was developed. This involved specifying the desired behavioural and environmental outcomes from the intervention in the target group (e.g. to take part in 120 min of purposeful walking per week). Next, the performance objectives (a description of what is required of the target group to perform the behavioural outcome or how environmental conditions will be modified) for each desired outcome were specified in a list-wise fashion (e.g. developing the intention to walk). This leads to the creation of a behaviour change matrix which specifies change objectives (i.e. what needs to change in order for the performance objectives to be met). Using the previous example, if a performance objective is “to develop the intention to walk”, a change objective might be “to know the health benefits of walking”. The matrix combines the evidence- and theory-based determinants of the desired outcomes that were identified in the needs assessments. The change objectives allow for the mapping of the performance objectives to practical strategies that encourage behaviour change.

### Step 3: Selecting theoretical methods and practical strategies

Step 3 involved choosing theoretical change methods on which to design the intervention and, from this, translating change objectives into practical strategies, which can underpin intervention components. Theoretical methods and practical strategies were chosen after a process of consulting with the relevant literature and findings from interviews in Step 1, engaging with the guidance given by Bartholomew et al. [18] and discussions with the planning group and PPI group.

### Step 4: Intervention programme production

In Step 4, the intervention materials and protocols were designed which included defining the scope and limitations of the intervention programme production and delivery. Materials were presented to and discussed with the PPI group to ensure that they could be refined appropriately and were user-friendly. The members of the PPI group were asked to complete a short questionnaire (Appendix C) exploring potential facilitators and barriers to intervention participation and what the indicators of a successful intervention might be. Based on their feedback, key changes that were feasible were made to the intervention components and materials.

### Step 5: Implementation and adoption plan

Step 5 required the planning group to address how the intervention could be transitioned from its theoretical state to the real world, thus considering how it would be implemented and by whom [18]. In this case, the intervention was to be implemented within a feasibility trial. Therefore, intervention execution was discussed in terms of a short-term adoption plan, as it was likely that the intervention would be refined for future long-term adoption.

### Step 6: Evaluation

Finally, the planning group developed a plan for evaluating the feasibility and acceptability of the intervention, based on the IM protocol and feasibility evaluation frameworks provided by the NIHR [24]. In turn, this plan will evaluate the results of the IM protocol steps.

## Results

### Step 1: Needs assessment

#### Rapid review of the literature

The small body of literature surrounding PA in the context of cervical cancer survivorship mainly focuses on whether survivors take part in PA [25], self-reported PA levels (e.g. [26]) and the relationship between PA and other outcomes such as QOL, fatigue and long-term survival (e.g. [27]). Two studies investigating the effect of a PA intervention in gynaecological cancer survivors, with some representation of cervical cancer, were found and are presented in Table 1. Nine studies were found which provide insight regarding PA preferences of gynaecological cancer survivors (endometrial, uterine, ovarian, cervical), which used either a cross sectional or qualitative study design.

**Table 1:** Physical activity intervention studies in gynaecological cancer, with some representation of cervical cancer

Author and country	Population	Key measures	Study design	Findings and evaluation
Yang et al. (2012) Republic of Korea [15]	34 gynaecological cancer survivors (93% cervical cancer) treated with radical hysterectomy and pelvic lymph node dissection Mean age = 52.4 years	Pelvic floor strength (MEPs); The pelvic floor questionnaire; QOL (EORTC-QLQ-C30; EORTC-QLQ-CX 24)	RCT; intervention (4-week pelvic floor muscle training programme [PFRF]; n = 17) and usual care (Non-PFRF; n = 17)	Completion rate = 86% Sig. improvements in the PFRF group in sexual function from T0-T1 Improvements in physical function, pain, sexual worry, sexual activity and sexual/ vaginal activity from T0 to T1
Donnelly et al. (2009) UK [14]	33 sedentary gynaecological cancer survivors (stage I–III; ≥ 3 years post diagnosis), experiencing treatment-related fatigue (12% cervical cancer) Mean age = 53 years	Fatigue (MFSI-SF; FACIT-F) QOL (FACT-G); depression (BDI-II); affect (PANAS); physical functioning (12-min walk test)	RCT; 12-week moderate-intensity PA intervention (PA; walking; strengthening exercises; weekly consultation; 2-follow-up telephone calls; n = 16) or contact control (CC) group (n = 17)	Recruitment rate (of those eligible) = 25% Sig. decrease in fatigue (P/I; F/U) and increase in QOL (F/U) in PA group compared to CC group Sig. difference in positive and negative affect between groups

***KEY:*** *n*, number of participants; *QOL*, quality of life; *EORTC*, European Organisation for Research and Treatment of Cancer; *MEPs*, motor evoked potentials; *MSFI-SF*, Multi-dimensional Fatigue Symptom Inventory–Short Form; *FACT-G/En*, the Functional Assessment of Cancer Therapy-General/endometrial cancer-specific scale; *BDI-II*, Beck Depression Inventory; *PANAS*, positive and negative effect; *Schedule*; *RCT*, randomised control trial; *PA*, physical activity; *Sig*., significant; *P/I,* post-intervention; *F/U*, follow-up; *T0*, baseline; *T1*,timepoint 1

#### PA preferences

The common types of PA performed by gynaecological cancer survivors are walking, gym-based activities and swimming [28, 29]. Moderate-intensity walking was found to be a preference for those not meeting the current PA guidelines and who reported lower-income levels [30–33]. The evaluation of a PA programme for socio-culturally diverse endometrial cancer survivors found that women particularly enjoy exercise programmes which provide opportunities for social interaction and which lead to physical benefits in terms of pain attenuation [34]. Programmes which gradually increase walking; incorporate PA into the daily routine of participants; provide a variety of exercises; incorporate regular goal setting; deliver regular counselling and provide the opportunity to socialise with similar others are also favourable [29, 33–36].

#### Interviews with women who have been treated for cervical cancer

Participant characteristics, treatment details and details of PA participation are included in Table 1. Participants enjoyed walking alone or with others, in contrast to class-based activities, which they perceived to be challenging to replicate alone.

Challenges and facilitators to PA most commonly spoken about are shown in Table 2. Challenges tended to be unique for each participant. Physical after-effects of treatment act as challenges either physically or by contributing to a psychological factor which hindered participation. For example, neuropathy affected balance, which in turn impacted one’s competence and confidence to try certain activities (e.g. cycling). A lack of knowledge of safe activities to undertake and environmental factors (e.g. requiring access to toilet facilities) were also challenges. Regarding facilitators, women were more likely to be active if they were aware of the benefits of being active and could incorporate being active into their daily routine. Enjoyment of PA was important and could be enhanced by setting small goals, tracking self-improvement, a sense of competition and being active with others who have similar experiences.

**Table 2:** Participant characteristics, treatment type and physical activity

	Age (yrs)	Treatment type	Time since treatment (months)	Physical activities post-treatment
1.	46	Radiotherapy, chemotherapy, brachytherapy	36	Aerobics, running, toning, walking
2.	38	Hysterectomy, radiotherapy, chemotherapy, brachytherapy	22	Walking, gardening, cycling
3.	49	Hysterectomy, lymph node removal	28	Dancing, yoga, Pilates, running, strength training
4.	37	Hysterectomy	58	Gym, walking, cycling
5.	34	Hysterectomy	32	Yoga, combat, boxing, interval/strength training, walking
6.	50	Radiotherapy, chemotherapy, brachytherapy	48	Walking, gardening
7.	44	Hysterectomy, lymph node removal	109	Cycling, walking
8.	33	Radiotherapy, chemotherapy, brachytherapy	42	Dancing, acrobatics, gym
9.	37	LLETZ, cone biopsy	84	Walking, running, gym, strength training, dancing, childcare
10.	37	Radiotherapy, chemotherapy, brachytherapy	2	Walking, dancing, stretching, jogging

Key: *LLETZ*, Large Loop Excision of the Transformation Zone. NOTE: All participants were White British

### Step 2: Identification of outcomes, performance objectives and change objectives

The National PA recommendations for adults are at least 150 min of moderate-intensity aerobic PA per week in combination with strength training [37], whilst PA recommendations for cancer survivors as suggested by the American College of Sports Medicine are at least 30 min of moderate-intensity activity, 3 times per week for at least 8–12 weeks [38]. Based on the data collected in Step 1, it was decided that the desired behavioural outcomes of the intervention were the adoption and increase of PA levels among the target group (those previously treated for cervical cancer and currently not taking part in 150 min of moderate-intensity activity per week) with a view of reaching the recommended weekly PA guidelines for aerobic activity. The chosen avenue for PA was walking as it is an activity which is specifically recommended for cancer survivors [38], with numerous health benefits documented such as reduced fatigue [14] and reduced cardio vascular risk [39]. It is an activity which is flexible and overcomes the many PA challenges faced by cancer survivors. For example, it places little pressure on the pelvic muscles which can be weakened as a result of treatment (e.g. [40]). Additionally, its intensity and duration can be adjusted to suit individual preferences and can promote social interaction by “walking and talking”.

Next, performance objectives and the behaviour change matrix were constructed, and the theoretical determinants involved in changing behaviour for each objective and the resulting performance objectives were identified (e.g. self-efficacy, knowledge, intrinsic motivation). Examples of performance objectives, theoretical determinants and change objectives can be found in Appendix D.

### Step 3: Selecting theoretical change methods and practical strategies

The social cognitive theory (SCT) [41] was selected as relevant for this intervention as many of the theory’s determinants (e.g. self-efficacy, outcome expectations, outcome expectancies, self-efficacy, behavioural capability and observational learning) [18] hold relevance for this intervention and have established theoretical change methods. The health belief model (HBM) and theories of self-regulation also informed intervention development.

During several planning group meetings, theoretical change methods were translated into practical strategies to directly manipulate elements of behaviour and the environment. Strategies were created based on what would be deemed feasible, given the population characteristics and previous successful strategies from relevant literature [42]. Examples of how theoretical methods were translated into practical strategies for this intervention can be found in Appendix E.

### Step 4: Producing an intervention programme plan

In Step 4, a structured intervention programme was developed by the planning group and incorporated suggestions from the PPI groups on the content, materials and time scales for the intervention. Scope and limitations of the intervention were identified. These were budget restrictions and the challenges as a result of COVID-19 restrictions on social distancing and household mixing. Whilst group walking was seen as the most effective strategy to encourage PA participation after cervical cancer treatment, it was not possible to make this a condition of the intervention as it needed to be flexible in order to be continued during possible restrictions imposed in the future.

The intervention programme to increase walking following treatment for cervical cancer contains structured components, which also provide flexibility and the opportunity for individualisation. The 12-week intervention will target individual determinants of behaviour change, with a focus on the practical strategies outlined in Table 3. The strategies are delivered in six related components of the intervention:

**Table 3:** Challenges and facilitators to PA participation identified in qualitative interviews

Barriers	No. of participants	Facilitators	No. of participants
Physical side effects of treatment		Feelings of competency	
I get hot and sweaty when I exercise (menopausal symptoms)	4	I know how to be prepared if I have an accident	2
I lose my balance easily (neuropathy)	2	I was an active person before treatment	7
Pain and heaviness from lymphoedema limits the types of activities that I can do	2	I feel less embarrassed if I am open about my issues	4
Psychological challenges		Enhancing enjoyment of PA	
I don’t feel confident in my body since treatment	5	I try to set myself targets and track my progress	9
I don’t have the same energy or motivation to be active as before	6	Being competitive with myself and/ or others	6
I fear that doing physical activity will make my symptoms worse	4	I feel like I have achieved something after I am active	6
A lack of knowledge		Structure/ routine	
I don’t know what physical activities my body can do	7	I like getting into a routine of being active	4
The environment		Knowledge of PA benefits	
I feel vulnerable walking in my neighbourhood	2	Being active helps to reduce my anxieties	6
There aren’t always access to toilet facilities	3	I feel like I have control over my health if I am active	8

*KEY*: *PA*, physical activity

#### Intervention launch and brief education

First, all participants will attend a virtual intervention launch session designed to increase knowledge on the benefits of walking for those diagnosed and treated for cervical cancer (education provision), facilitate group discussions on addressing perceived challenges to taking part in PA and consider the benefits of goal setting (and how to set goals) to gradually increase PA.

#### Physical activity self-monitoring tool

Each participant will be provided with a Fitbit activity monitor to support self-monitoring of their PA and to provide PA prompts and prompts to review goals throughout the intervention. Self-monitoring, via feedback, is associated with PA behaviour through promoting self-efficacy for PA [43] and is accepted after gynaecological cancer [44].

#### An intervention diary

A handwritten diary with daily and weekly inputs will facilitate assessment and evaluation of physical and psychological well-being. The diary is an opportunity for self-reflection to help participants to gradually build confidence in their own capabilities.

#### Health coaching

Health coaching will be offered to participants fortnightly via telephone or video call. Health coaching sessions will be delivered by NM who has MSc level training in exercise psychology and informal training supported by all authors who have various health coaching qualifications, of which FM has used in her cancer research. The sessions will follow a schedule, based on the GROW model [45] to guide individualised barrier identification and problem solving, goal review and goal setting to increase PA.

#### A messaging platform

Group messaging between participants on the Fitbit community or via WhatsApp will act as a platform to encourage peer support and to organise a time and a place for group walks (if possible), whereby participants can state their intention to be active and hold themselves accountable (implementation intentions).

#### Group walking

Providing the opportunity for participants to be active together will create a supportive environment which fosters feelings of relatedness and identification with similar others which have been linked with enhanced PA enjoyment [46].

It was chosen to deliver intervention components on virtual and technology-based platforms where possible to allow for individualisation and to enhance adherence. For example, education and health coaching will be delivered on the Microsoft Teams platform. The intervention will be delivered in groups of up to six participants who live in relatively close proximity to each other, to facilitate group walking.

### Step 5: Implementation and adoption

The planning group agreed that the intervention should be implemented within a feasibility trial. Discussions around the intervention programme identity with the PPI groups led to the name ACCEPTANCE (Acceptability in Cervical Cancer of an Exercise-based Programme delivered Through An Online Community Environment).

Six months post-cervical cancer treatment was chosen as an appropriate criterion for inclusion in the intervention study. The qualitative findings suggest that it is a time when patients feel ready to resume normal activities. Previous research suggests that gynaecological cancer survivors have a preference for PA after treatment rather than at diagnosis or during treatment [30] and that between 6 and 9 months post-treatment is a period of heightened motivation for positive change, therefore presenting a critical time for intervention [29, 47]. In terms of reach, it was decided that clinical nurse specialists would be engaged and trained to offer the intervention programme to patients who attend clinic. For those who have been discharged, relevant cancer charities would be engaged to promote the intervention programme.

### Step 6: Evaluation plan

An evaluation plan to test the feasibility of the PA intervention programme was created to gain insight into the acceptability of the intervention components and to determine whether refinement was needed in order for the intervention to be suitable as a full-scale pilot trial. Evaluation time points will be at baseline, 6-week, 12-week and 3-month post-intervention (24 weeks). The process evaluation methods will be a set of 3 online questionnaires designed to obtain feedback on participant experiences of attending the programme launch session, of using the Fitbit and of participating on the messaging platform; follow-up interviews to gain insight into how participants experienced the intervention; device assessed PA data via 8 days of wearing an accelerometer at all 4 time points; and a record of participant feedback throughout the intervention.

## Discussion

The IM protocol provided the planning group with a structured and systematic approach to intervention development. The planning group also benefitted from the feedback given by the PPI group who represent the target population for the proposed intervention. The integration of theory is at the centre of this protocol which is in line with the recommendations offered by the Medical Research Council [16] and scholars specialising in behaviour change [48]. Based on previous literature and findings from the interview study, SCT was chosen as a main theory which incorporates self-efficacy along with input from the HBM and theories of self-regulation. Among cancer survivors, perceived self-efficacy in undertaking PA is positively associated with increased PA behaviour [49]. More specifically, SCT has been used to design and develop feasibility and longitudinal PA interventions for endometrial cancer survivors [50]. Practical strategies were also theory-driven; for example, technology was chosen as a key strategy to promote self-monitoring behaviour, self -efficacy for PA, implementation intentions and to provide choice and flexibility within the intervention. Technologies, such as the Fitbit, are a widely accepted and integrated facet of society and have been utilised to increase PA behaviour after cancer [51]. Cancer survivors have previously accepted technology-based methods for enhancing social support, instruction via coaching and self-regulation behaviours [33].

Given that exploring cervical cancer survivorship is a novel avenue in the PA research landscape, assembling a PPI group to advise the development of this intervention along with findings from the interview study was highly valuable. A key assumption of this research is that behaviour change techniques need to be specific to those treated for cervical cancer. Regular contact with the PPI group enhanced this focus and ensured that all decisions were made with respect to those who would receive the intervention. Such insights allowed the planning group to build a unique knowledge base regarding the experience of recovery which was essential when choosing theoretical determinants to target behaviour.

The qualitative findings echo previous data suggesting that gynaecological cancer survivors do associate PA with cancer-specific health benefits; however, treatment side effects act as a significant barrier [33]. In this study, we found that participants enjoyed home-based walking and gym activities and welcomed exercising with other survivors who had similar experiences. Such findings are both supported and contested in the literature with some research suggesting that cancer survivors do not enjoy partaking in lifestyle interventions with other survivors [30], hence further highlighting the need for interventions tailored by cancer type, particularly when cervical cancer survivors generally represent a younger demographic compared to more commonly researched populations (e.g. breast cancer survivors). A limitation of the qualitative study was that it lacked diversity in terms of PA participation. All interviewees identified as taking part in some physical activity, which contradicts the current literature [52]. However, this may be due to the study explicitly stating its interest in PA during recruitment. The intervention strategies aim to increase PA behaviour in those who are not physically active, and so it would have been meaningful to also speak with women who were not active to gain a greater insight into further possible barriers experienced. Greater diversity in recruitment could have been achieved had it been possible to recruit via hospital clinics.

Whilst the protocol provides a structured and systematic framework to base intervention development, the level of detail required to complete each step leads to an onerous process. Previous researchers have commented on the time-consuming nature of the IM protocol [21]. The protocol may not always be a feasible option when developing clinical interventions as such studies require more time-consuming ethical clearances and funding. It took the planning group 8 months to complete the IM process, and a further 7 months to obtain ethical and regulatory approval. These two processes cannot happen simultaneously because the exact protocol needs to be confirmed before the study can be submitted to the NHS ethics committee.

The protocol was suited to the development of a feasibility trial. However, adoption and implementation of the intervention (Step 5) will have to be revisited should a full-scale trial be implemented as the considerations made here will differ to those made in preparation for a feasibility trial. In terms of feasibility evaluation, it was also necessary to combine another framework in order to develop an appropriate evaluation plan; in this case, the NIHR framework was used [24] as the IM protocol does not specifically deal with the evaluation of feasibility aspects.

## Conclusion

This paper maps the development of a physical activity intervention programme suitable for those recovering from cervical cancer treatment. The initial protocol steps informed what needed to be targeted by the intervention, whilst the latter steps allowed development of strategies and protocols to achieve this. Although it is an onerous process, intervention mapping is worthwhile if adequate resources are in place.

## Supplementary Information

ESM 1 (DOCX 26 kb)
