# Supplementary material for: Increasing physical activity levels following treatment for cervical cancer: an intervention mapping approach
Source: J Cancer Surviv. 2021 May 26;16(3):650–8. doi: 10.1007/s11764-021-01058-y (PMC8153850; doi:10.1007/s11764-021-01058-y)
Supplement: Supplementary file 1 — (DOCX 26 kb) [file 11764_2021_1058_MOESM1_ESM.docx]

**Appendices**

Title: Increasing Physical Activity levels following treatment for Cervical Cancer: An Intervention Mapping Approach.

**Appendix A**- List of search terms used in rapid review of the literature

The search used the following key words : for terms referring to cervical cancer survivors (“cervical cancer,” “cervical cancer survivor,”) for gynaecological cancer survivors, (“gynaecological cancer survivors,” “endometrial cancer survivors” Ovarian cancer survivors,” “Womb cancer survivors”, “vulvar cancer survivors”, “vaginal cancer survivors”), for physical activity (“physical activity,” “exercise,” “physical activity programme,” “walking” “Activity”) for intervention, (“Intervention,” “trial,” “Randomised control trial”) for PA preferences (“Physical activity preferences”, “exercise preferences”. Every term used for cervical cancer survivor or gynaecological cancer survivor was combined using “AND” with every term for physical activity and intervention. Every term used for cervical cancer survivor or gynaecological cancer survivor was combined using “AND” with every term for physical activity preferences.

**Appendix B-** Template analysis coding template

| **Theme** | **Sub themes** | **Codes** |
| --- | --- | --- |
| **Barriers to physical activity** | physical side effects of treatment | - Bowel issues/ diarrhoea - Urgency (Bladder) - Side effects of menopause - Energy levels - Neuropathy (loss of balance/ stumbling) - Pain as a result of a seroma - Lymphoedema - General Pain |
|  | Psychological barriers | - Lack of confidence to try new things - Lack of motivation (Weight loss not a motivation for PA) - Mental fatigue - Low feelings of competence |
|  | Lack of time | - Having other priorities |
|  | Environment | - Feelings of physical weakness - Unable to protect oneself - Lack of facilities |
|  | Fear of putting oneself at physical risk | - knowledge of capabilities - Knowledge of what is safe to do post treatment |
| **Facilitators to physical activity** | Knowledge/ Self Awareness | - Previous experience of PA - Information provision (PA and treatment effects) - Sense of identity through PA - Knowledge of PA benefits - Knowledge of capabilities |
|  | Feelings of relatedness | - Being open about issues (class-based situation) - Being held accountable for PA - Feeling normal - A source of motivation |
|  | Physical activity goals | - Small PA goals after treatment (e.g. Having a shower; walking to the shop) - Sense of achievement - Bettering oneself - Source of competence |
|  | Ability to be active/ competence | - Keeping track of physical activity - Competition with others and oneself |
|  | Knowledge of benefits / feeling in control of one’s health | - PA to alleviate anxiety - PA to help with emotional coping - Body confidence   Prevent recurrence   - Alleviate pain caused by lymphoedema - Weight loss - Feelings of strength |
|  | Physical activity being a part of one’s structure/ routine | - A sense of normality - Walking with others - PA as part of travel routine - Walking dog/ childcare |

**Appendix C-** Short questionnaire to obtain PPI feedback

| **1. If you heard about the study….** | a. What would attract you to the study? |
| --- | --- |
|  | b. What would stop you from doing the study? |
| **2. If you agreed to take part in the programme…** | a. What aspects of the programme do you think you would enjoy? |
|  | b. Are there any aspects of the programme you think you might struggle to engage with? |
| **3. What do you think would motivate women to do the evaluation measures?** | |
| **4. What do you think would have to happen for the programme to be deemed successful?** | |

| **Appendix D**- Examples of performance objectives, determinants, and change objectives for ACCEPTANCE | | |
| --- | --- | --- |
| **Performance Objective** | **Determinants** | **Change Objective(s)** |
| PO.1. Decide to increase PA levels | Self- efficacy | Identify situations in which you would feel confident doing PA; List types of PA you feel capable doing |
|  | Knowledge | Know what PA means to you |
|  | Health related benefits | List physical and psychological health benefits of partaking in PA |
| PO.5. Plan when and where the structured walking will take place | Self- efficacy | Express confidence in committing to a plan of PA; State times of days that you associate with PA |
|  | Knowledge | List the possible locations that you could walk for at least 30 minutes; |
|  | Health related benefits | State times of the day that physical activity could be most beneficial for you |
|  | Intrinsic motivation | State how making a plan will impact your motivation for PA |
| PO.6. Identify personal motivation for physical activity | Perceived self- efficacy | Identify why you chose to be active in the past; Express confidence in your reasons for wanting to be active now |
|  | Intrinsic motivation | List challenges to PA that you are currently experiencing and why you would want to overcome these |
|  | Self-regulation | Write down your reasons for being physically active at the start of every week |
| PO.8. Break national guidelines into manageable PA chunks in line with your weekly schedule | Perceived self- efficacy | Express confidence that you can create smaller goals from a large goal |
|  | Intrinsic motivation | State your preference for PA (duration and frequency) |
|  | Self-regulation | Keep track of the number of sessions you complete each week |
|  | Knowledge | Be aware of what PA opportunities are available to you |
| PO.16. Identify a mechanism for monitoring weekly physical activity | Self- efficacy | Express confidence in your ability to monitor your PA |
|  | Knowledge | Decide what types of PA information you would like to know; Seek information about different types of monitoring |
|  | Health related benefits | Monitor any changes in psychological health alongside PA |
|  | Intrinsic motivation | Explain how monitoring your PA will influence your motivation for PA |

**KEY: PO= Performance objective; PA=Physical activity**

| **Appendix E-** Change objectives, theoretical change techniques and practical strategies | | |
| --- | --- | --- |
| **Change objective** | **Theoretical change methods** | **Strategies** |
| Know that being physically active will be beneficial to you | Knowledge provision  Perceived benefits and barriers (HBM)  Reinforcement | Interactive education session between participants. Focused on PA.  Education reinforced via take home leaflet |
| Express confidence in ability to walk | Knowledge provision  Vicarious experience (SCT)  Past experiences (SE; SCT)  Barrier identification | Interactive barrier identification session  Self-reflection on previous experience  An opportunity to foster feelings of relatedness  Problem solving via health coaching |
| Plan to walk with others | Implementation intentions  Public commitment | Organising group walks via a messaging group  Instilling a sense of commitment via posting on messaging group |
| Have an awareness of the amount of physical activity you are doing; Have an awareness of improvements | Self- monitoring (SCT; SRT)  Self-reflection (SCT) | Self- monitoring of steps and minutes of PA  Self-reflection on well-being  Self-assessment of well-being and capabilities |
| Plan to increase physical activity during intervention | Implementation intentions  Self- monitoring (SR)  Goal setting (SCT)  Autonomy (SDT) | Outlining when and where PA goals will take place  Setting long term PA goals  Reflecting on PA to inform goal setting  Setting specific, short term, realistic PA goals |

*KEY:* HBM= Health Belief Model; SCT= Social Cognitive Theory; SE= Self- efficacy; SCT= Social Cognitive Theory; SRT= self- regulation theory; SDT= Self- determination theory
